# Counter Prescribing

**Published:** 1907-11

**Authors:** 


### EDITORIAL NOTES AND COMMENTS.

The Business Office of the JOURNAL-RECORD is 1 1-2 to 5 1-2 South Broad Street.

The Editorial Office is 318-319-320 The Prudential.

Address all Business Commucications to Dr. George Brown, Manager.

Make remittances payable to THE JOURNAL-RECORD OF MEDICINE.

On matters pertaining to the Editorial and Original communications, address Dr. Bernard Wolff, Atlanta.

Reprints of original articles will be furnished at cost price. Requests for the same should always be made on THE MANUSCRIPT.

We will present, postpaid, on request, to each contributor of an original article, twenty (20) marked copies of THE JOURNAL-RECORD OF MEDICINE containing such article.

### COUNTER PRESCRIBING.

Considerable bitterness has arisen between the physicians and druggist in Augusta, Ga., over the matter of "counter prescribing." The druggists are incensed that the doctors should question their right to prescribe, while the physicians think that it is useless for them to go to the expense of graduating in medicine if the privileges for which they have worked are to be allowed to pharmacists and others who are only familiar with the drugs and not with the diagnosis of the diseases for which they are prescribed.

Self-medication is undoubtedly an evil of considerable magnitude and far-reaching in its effect and should be discouraged wherever possible. The time lost in such treatment frequently is sufficient to allow the disease to progress from a simple malady amenable to appropriate measures, to a complicated condition which requires prolonged treatment and expense or is perhaps fatal on account of the delay. That it would impose a hardship on the poor who are not able to consult a physician for every slight ailment or "bilious attack," if they were not mit. There is "middle ground," however, which is undoubted-given simple remedies by the druggists, we are willing to ad-ly nearer right than either extreme, and the close relation between the physician and druggist should be fostered on the part of both to lessen friction where their respective privileges overlap.

An unfortunate and apparently unfair practice obtains among most druggists who charge a higher price for medicine when ordered in a prescription than when the same preparation is purchased by the patient without a prescription. This does not seem to be treating the doctor with due consideration.

Upon being questioned about the extravagant untruthful claims of preparations he was advertising, a local druggist said: "You doctors need not kick, as many of the patients who treat themselves bring on serious complications for which a larger fee can be charged than the primary condition would have justified." This is not dealing fairly with the patient.

